# Virtual patients and learning communication skills at “double speed”: a qualitative study using the TPACK framework

**DOI:** 10.1186/s41077-026-00481-4

**Published:** 2026-08-24

**Authors:** Criona Maria Walshe, Catherine Bruen, Ronan Daly, Fiona Kent

**Affiliations:** 1 Health Professions Education Centre, Royal College of Surgeons in Ireland, Dublin, Ireland; 2 Department of Obstetrics and Gynaecology, Royal College, Dublin, Ireland

**Keywords:** Virtual Patients, Technology, Communication Skills, Medical Students

## Abstract

**Background:**

Effective communication is central to patient-centred care, but achieving sufficient practice opportunities with access to expert feedback during busy clinical placements can be challenging. Virtual Patients (VPs) simulate doctor-patient consultations and have been used successfully in medical education to teach communication skills. Four VPs were created with filmed patient encounters, using a branching decision format and scripted expert clinician feedback, to provide students with opportunities to practise challenging, infrequently seen, communication encounters.

**Methods:**

One focus group and six individual interviews were conducted with medical students as a quality improvement measure prior to the implementation of VPs into the year 4 medical curriculum. After transcription, deductive thematic analysis was undertaken using the TPACK framework as a conceptual lens to examine technological, pedagogical, content and contextual knowledge.

**Results:**

Students described the VP content as authentic, emotive and supportive of their clinical, communication and cultural learning. They reported enhanced confidence through scaffolded exposure, but emphasised VP inferiority to real-world patient encounters. Pedagogically, students learnt through observation of videos modelling good communication under challenging circumstances, such as communication of a miscarriage, and suicide risk assessment following self-harm. However, students tended to skip or minimise VP reflective tasks, and they described the automated feedback as didactic and non-memorable, limiting educational impact.

Students’ technology expectations exceeded the platform capabilities. They sought to speed through sections where possible and the VP activity was described as “clicking”, rather than communicating. Despite these limitations, students gained contextual knowledge and suggested the integration of VP into the curriculum as preparatory learning rather than communication skills practice, as they recognised that despite enhanced confidence, mastery requires repeated engagement with real or simulated patients.

**Conclusions:**

This VP platform did not meet the needs of students to practise challenging communication skills, with technology insufficiently integrated to align content delivery to pedagogical intent. Our findings suggest that although useful to complement live or simulated patient communication encounters, to realise their potential, VP technologies must enable more authentic interactivity such as voice-to-text, nuanced individualised feedback and stronger alignment between TPACK domains.

**Supplementary Information:**

The online version contains supplementary material available at https://doi.org/10.1186/s41077-026-00481-4.

## Background

Excellent communication skills are prerequisite to becoming a competent healthcare professional, with strong evidence that effective communication positively impacts patient satisfaction [1–3], adherence to treatment regimens [2, 4], patient outcomes [5] and doctor satisfaction [6]. Communication skills are a tangible entity that can be taught [7], learned [8], and assessed [9]. However, communication skills are complex and require effective education programmes, with opportunities to practise, engage in reflection and receive feedback [10]. Despite this, wide variation persists in communication skills curricula [11, 12], teaching modalities [13], and assessment approaches across medical schools.

Clinical placements seek to provide sufficient opportunities for communication skills development, however, optimal teaching in the clinical learning environment is constrained by service pressures and uneven access to diverse patient groups. Virtual patient consultations [VPs] are an innovative technological approach that may address these challenges. VPs are “*interactive digital simulations of clinical scenarios for the purpose of health professions education”* [14], and have been used with success in a variety of contexts to teach and develop communication skills, including healthcare [15].

At RCSI University of Medicine and Health Sciences in Ireland (RCSI), VPs have been introduced into the medical curriculum to enhance communication skills training. In 2025 we developed four new VPs in an interactive video-recorded platform, to address complex communication challenges to which students may not otherwise gain exposure. Scripts were co-designed with subject matter experts and a patient advocacy group to reflect authentic real-world case complexity. Separate scripts were developed for both the virtual patients and their family and carers, and the avatar coach in each VP who provided expert clinician feedback at each decision point.

The Technological Pedagogical Content and Knowledge (TPACK) framework conceptualises technology-enhanced learning as a dynamic interplay between three domains - content knowledge, pedagogy knowledge and technology knowledge [16, 17]. TPACK asserts that these domains must be carefully integrated to create effective educational interventions, positing that the interplay between them is as important as each domain itself. In this exploratory qualitative study, we explored student experiences and perceptions of VPs, through the lens of the TPACK framework. The research sought to contribute new insights into the value of VPs to support communication skills development among medical students.

## Methods

### The innovation

The undergraduate medical curriculum was blueprinted against two communication skills frameworks [18, 19], to establish communication skills practice opportunities for Year 4 medical students. Four VPs were developed to align to the specialties through which Year 4 students rotate: Obstetrics and Gynaecology, Psychiatry, Paediatrics, and a combined Medicine and Surgery placement. Communication learning outcomes for each VP were aligned with module outcomes, providing structured practice opportunities for communication challenges. Case complexity was aligned to Year 4 student level to avoid cognitive overload [20]. The Obstetrics and Gynaecology VP focussed on miscarriage counselling and aimed to model best practice when working with an interpreter. The Paediatric VP focused on anaemia in a child from a marginalised ethnic minority, aiming to build cultural awareness and address racism and bias. The Psychiatry VP required a suicide risk assessment of a single mother following an episode of self-harm, which had been witnessed by her children, requiring understanding of child safety legislation. The Medicine/Surgery VP explored history taking from an older person with cognitive impairment and modelled a telephone call with a community pharmacist. High quality video footage of simulated participants was created to portray the virtual patient, family or caregiver.

Within each VP, students assumed the role of a doctor undertaking a patient consultation. Each VP followed a branching pathway, with students selecting from three text options at each decision point (optimal, suboptimal and critical). Deviation from these text options was not possible, with no free-text or voice-to-text input options available. Having made their text selection, students viewed the virtual patient’s reaction to their choice, prior to moving to the next decision point. Coaching feedback was delivered by an avatar coach created using Synthesia, an AI video-generation platform [21]. All media were edited and uploaded into the VP platform which was hosted on the learning management system (Moodle). A screenshot of the platform is included in Appendix 1. Each consultation began with a brief introduction by the coach, and a patient referral letter which provided students with context and background information. Recommended resources were provided as pre-reading (e.g. link to previous lecture and clinical practice guidelines). Reflection tasks and “end of case” summaries (e.g. writing a letter to patient’s GP) were embedded to maximise learning [22].

### Study design and participants

This study used an exploratory qualitative design employing one focus group and six individual interviews. Questions were semi-structured and aligned to TPACK domains. This approach facilitated in-depth exploration of how students experience the interplay between the four TPACK domains. An advertisement was placed on Moodle, inviting Year 3 and Year 4 medical students to trial the new VPs and provide detailed feedback before curricular implementation.

Ethical approval for the study was provided by the RCSI Ethics Committee, Approval ID 50556.

### Theoretical framework

TPACK underpinned study design and data analysis [16]. TPACK was developed to describe how a teacher could successfully integrate technology into education, to support student learning [17], so we sought to understand how that intended interplay was experienced by students. The TPACK framework was updated in 2025 to incorporate contextual knowledge, reflecting the importance of educational setting on learning [23]. This domain was explicitly integrated into the data analysis to evaluate how the educational context shaped student perceptions.

### Data analysis

The focus group and interviews were held online, audio-recorded and transcribed verbatim. Data were analysed using deductive thematic analysis, guided by the TPACK theoretical framework. The six phases of thematic analysis were followed, incorporating familiarisation, generation of initial codes, searching for themes, reviewing potential themes, defining and naming themes and generating a report [24]. Two researchers (CW and FK) independently coded transcripts, then met to share findings, discuss and name themes by consensus.

## Results

Following informed consent to participate in the study, one focus group comprising three students, and six interviews were convened via video conferencing software during June/July 2025. Focus group speakers are identified as FGP1-3, interviewees are identified numerically as Interviewee 1–6. Themes raised under each TPACK domain and definitions of each theme identified are summarised in Table 1.

**Table 1:** Themes raised under each TPACK domain, and definitions of themes

Domain	Themes	Definitions
Content	Clinical and cultural Knowledge	Clinical and cultural learning outcomes are achieved through authentic communication challenges
	Increased Confidence	Scaffolded learning addressing clinical interactions students would not otherwise have had access to, builds confidence as students feel more prepared for clinical practice
	Learning about communication through emotive scenarios cannot replace real-world experience	Emotive cases generate emotional responses in learners enhancing engagement as learners learn about communication skills however this cannot replace real-world experience
Pedagogy	Learning through modelling	Students see what good communication looks like, and learn as a result, often considering how they will build what they have seen into their own clinical practice
	Autonomous navigation	Navigating through the VP is driven by efficiency rather than pedagogical intent as students seek to speed up videos, skip pre-reading, tasks, feedback, even when they know it is of value. If tasks are not mandatory, they will not be completed
	The Coach - Not individualised feedback	Although some students found value in the coach, others found the feedback didactic and non-memorable. Requests to speed up and skip suggest that many did not see the feedback as pertaining to their individualised performance
Tech	Expectations are high	Student expectations of educational technology are high and the platform fell short of these
	Predetermined text dialogue	Predetermined text-based options reduce the complexity of inter-personal communication
	Clicking, not communicating	Students describe their actions with the simulation as “clicking” rather than communicating suggesting the platform does not offer communication skills practice.
Context and Contextual Knowledge	Students will not engage unless mandatory	Students describe their context, time pressures and do not have capacity to engage with non-mandatory curricular elements
	Lack of transferability	Students acknowledge limitations of VP and their limited transferability to real-world context
	Alternative contexts for use	These VPs do not provide communication skills practice, but are useful, and could be used as a preparatory resource or adjunct to alternative educational activities

### Content domain - what students learned

#### Clinical and cultural knowledge

Students perceived the VPs as authentic representations of challenging clinical consultations and described learning about diverse cultural groups and social determinants of health.

*“I learned a lot about the Romani community that I didn’t know*,* like I didn’t know that Romani was not a written language*,* and you have to find other ways to talk to your patients or to communicate with them basically.” (Interviewee 1*,* Female)*.

*“It was a really good exposure to real life situations …… they have a lot of distrust or mistrust in the medical community.” (Interviewee 5*,* Female)*.

Students’ reflections indicated that the intended clinical and cultural learning outcomes were largely achieved.

#### Increased confidence

Students reported increased confidence through structured exposure, observation of modelled interactions and reflection opportunities, particularly in scenarios they would not otherwise encounter in their medical education. Several students felt better prepared to attempt the communication challenges in future real-world practice.

*“I think they’re good for situations you might not have been in*,* like difficult conversations like the miscarriage or using an interpreter and how you would approach that” (FGP1*,* Female).*

*“I’ve got the basics of it now. I’d say I would know how to run through the scenario if I had to do it in intern year or whatever.” (Interviewee 4*,* Female)*.

#### Learning *about* communication through emotive scenarios cannot replace real-world experience

While students valued the emotionally challenging nature of the VPs, they consistently framed the learning as being ***about*** communication and highlighted that VPs cannot replicate the learning achieved through meeting real patients.

*“Because nothing beats being like face to face with people and I just don’t think you can ever really recreate that.” (FGP2*,* Female)*.

*“I feel like it’s good for learning information*,* but I don’t know if they’re any good for communication skills.” (Interviewee 1*,* Female)*.

### Pedagogy domain - how students learned

#### Learning through modelling

Students experienced the VPs primarily as a modelling resource, as they described learning from watching clinicians approach difficult conversations, handle sensitive moments, use non-verbal language and demonstrate empathy. Students outlined their intentions to apply this knowledge in their own future practice.

*“it’s really nice to see how interpreters should be used.” (FGP1*,* Female)*.

*“ I would be able to replay the video in my head and be like so this is how and this how the actors approach it and this is like how I should approach it and be professional” (Interviewee 4*,* Female)*.

#### Autonomous navigation

Navigation through the platform appeared to be driven by efficiency rather than pedagogical intent. Students described “clicking” through, and skipping or bypassing optional pre-reading, reflective tasks and coach feedback to advance through the activity with speed rather than full engagement. Although some did engage with the end-of -case reflective task and found this useful.

*“I didn’t even bother filling in anything…. I didn’t even stop to listen to the coach so maybe you guys could do something like you absolutely have to listen to the thing before you can go to the next one coz I found it really easy to just like select an option and just like skip through it” (FG2P1)*.

*“Paeds*,* that was good but I didn’t do the pre reading it had given me… I just skipped through the whole thing coz I realized that you could just like click the answers you thought were right and then just move on…” ( Interviewee 3*,* Female)*.

#### The coach - not individualised feedback

Perceptions of coach feedback were mixed. While some valued the explanations, others found the coach feedback didactic, focusing on “right or wrong”. Their requests to speed up and skip feedback sections, suggest that the one-directional feedback was not deemed useful for their individual needs.

*“I couldn’t see if it was wrong what was wrong with it*,* there was no sort of like explanation telling me what was that” (FGP1)*.

*“she wouldn’t tell me that it was wrong she would be like good try and then she would go on and on by the end of it she would be like oh you failed and I would be like “what” I thought you said that I did good.” (FG2P1*,* Female)*.

### Technology domain - how the platform shaped the learning experience

#### Expectations are high

Students expected more sophisticated functionality from the technology including playback speed controls, facility for non-linear navigation, ability to log off and resume at the previous point. They also found the progress bar indicating their advancement through the VPs insufficient.

*“Maybe having a good option to speed it up would be good*,* because I feel like everyone watches their lectures on two time speed these days.” (Interviewee 5*,* Female)*.

*“Ideally what would be nice would if you could go back to the start of the video because what I found was*,* I was going through scenarios yesterday and I picked up one of them but I had already started it and I wasn’t able to go back to the beginning” (Interviewee 2*,* Female)*.

#### Predetermined text dialogue

Students expressed frustration with the three fixed text-based options at each decision point, noting how different the options sounded when spoken compared with what they anticipated based on the written text. Students felt this constrained their responses and shifted focus toward choosing the “correct answer”, rather than making considered communication choices.

*“It sort of just felt like I was more so trying to choose the right answer instead of actually responding to the patient” (FGP3*,* Female)*.

*“Because you’re reading the options and when you’re speaking it out loud it’s different. Like when you’re reading it it’s like oh this is the right amount of information but it actually takes like a minute and a half to read all that out loud and then you realize oh my god that might be a bit overwhelming for the patient” (FGP2*,* Female)*.

#### Clicking, not communicating

The platform did not provide authentic communication practice opportunities as evidenced by student descriptions of “clicking” rather than communicating through the VP. Students also commented on their inability to demonstrate non-verbal language, and noted the lack of reciprocal patient responses.

*“Maybe if we could have like a speaking aspect of it. … If it could be like an actual patient if you were talking to them and they were talking back to you… on your response.” (Interviewee 1*,* Female)*.

*“you’re sort of just clicking the responses instead of actually really responding to a patient in the sense that you wouldn’t really be able to show the non-verbal aspects of it like giving the patient space*,* handing them a tissue or leaning forward to them and eye contact and all that stuff. I feel like all that isn’t*,* like*,* you can’t really do that when it’s a virtual patient” (FGP3*,* Female)*.

### Context and contextual knowledge

Students emphasised contextual constraints such as assessment priorities and time pressures, which shaped engagement with optional activities such as VPs. Students felt that the VPs will not be widely used unless mandatory, or credited.

*“I think they are useful*,* but when I heard about them first*,* I was like oh here is another thing we just have to do*,* to tick off the list” (Interviewee 2*,* Female)*.

*“We don’t do anything*,* nobody does anything*,* unless it’s compulsory. I definitely think like just having something…like completing it gives you a grade toward your GPA.” (FGP2*,* Female)*.

Students demonstrated contextual knowledge in recognising the limitations of the VPs in terms of transferability to real-world scenarios.

*“I feel like virtual patients or simulation that’s virtual better for learning clinical context rather than learning how to communicate with a patient” (FGP3*,* Female)*.

Students acknowledged the limitations of VPs as a communication skills training mechanism, suggesting use instead in alternative contexts such as a preparatory resource prior to meeting patients, or as an adjunct to other educational activities.

*“.if we were doing actual actors and a tutor with us to do these kind of scenarios these would be really good to do before in the days leading up to that kind of prepare you to kind of get you in that mindset I think that would be a really good use for them.” (FGP2*,* Female)*.

## Discussion

The TPACK framework recognises that *“particular technologies have their own propensities*,* potentials*,* affordances*,* and constraints that make them more suitable for certain tasks than others”* [17], and so is well suited to the aims of the current study. Our exploratory findings, framed through the TPACK lens, indicate that although students experience meaningful learning about communication through engagement with the VPs, the technology used in the current study did not sufficiently support the underlying pedagogy. This misalignment contributed to student perceptions that these VPs did not provide communication skills practice opportunities, although we acknowledge that this may not be the case in alternative VP platforms. Students described learning about communication through modelling, however the experience of these VPs was perceived as “clicking” rather than communicating. At the intersection of domains, this misalignment becomes more pronounced. Specifically, at the Content-Pedagogy-Technology intersection, the technological constraints of the platform limited engagement with culturally complex cases, to modelling and observation, rather than facilitating authentic communication practice. As a result, students experienced the communication learning primarily at cognitive and emotional levels rather than at a performance level. At the Content-Technology intersection, although students perceived the cases as realistic, the interaction did not feel authentic, as the technology limitations meant students focused on selecting the “correct” response rather than making considered communication choices, reducing complex interpersonal communication to a task-completion exercise. At the Pedagogy-Technology intersection, autonomous navigation undermined pedagogical intent as the “shortcuts” afforded by the technology, such as the ability to skip tasks or speed up videos, reduced reflection and deeper engagement.

Many advantages to VPs have been proposed in the literature, including the ability to practise new skills in a safe environment without fear of harm to patients [25] or shame to the learner. There is also the ability to utilise the technology for learning at any time and repeatedly [26]. Studies evaluating learner experiences of VPs align to this, as students describe VPs as motivating [27], safe environments to make and learn from mistakes, and build confidence [28]. Yet, despite these proposed advantages, clear evidence of benefit has not been demonstrated. In a recent review evaluating VPs used to develop clinical reasoning skills, benefit was only seen in 11 out of 19 studies [29]. Although Kononowicz et al. [14] found evidence of improved skills with VPs when compared with traditional education in their review of 51 trials evaluating VPs, the evidence levels were low to modest. Furthermore, interpreting the evidence around VPs is challenging, as, despite guidelines regarding the need to provide clear description of medical education interventions [30, 31], and proposed VP classification systems [32, 33], the literature lacks clear description of the technology used and the competencies achieved. There would be merit in an ethnographic observation of ‘how’ medical students engage with VPs, given the reflections observed by our cohort.

Across the literature, evidence of improved communication skills through training with VPs is mixed. Although some studies evaluating learner perspectives identified improved communication skills [34], others did not. Participants in one qualitative study stipulated that the development of communication skills “falls out of the scope” of VPs [28]. In another study, although participants noted progression in their communication skills, they outline the limitations of the virtual environment with regard to emotional connection and non-verbal communication [35]. In their systematic review, Lee et al. found half of eight comparative studies demonstrated improvement in skills, and conclude the best use of VPs is when aligned with other educational interventions [36]. In their recent scoping review, Kelly et al. [20] evaluated 22 VP studies with communications skills as the intended learning outcome and found varied outcomes. They conclude by highlighting the importance of reporting not just the VPs, but also curricular context.

Students in this exploratory study reported enhanced confidence and preparedness for practice, valuing exposure to emotive, authentic scenarios they would not otherwise encounter. This aligns with the findings of others who describe how emotive authentic cases enhance learning [28]. However, although our results demonstrate educational and experiential benefits, as students describe valuable learning *about* communication, it is more difficult to establish any potential long-term impact on student attitudes or behavioural changes. The creation process for high-quality VPs is resource-intensive [22] and our findings question the justification for such an undertaking. Further research is required with the more recent talk-to-text VP software now on the market, in order to ensure there is sufficient return on investment.

The importance of non-verbal communication cannot be overstated [37], and the inability of VPs, and education technology more broadly, to provide non-verbal communication training is well established [36]. Bearman et al. noted that although VPs that use a “narrative” pattern were more effective than “problem solving” patterns at teaching communication skills, the skills achieved were limited to verbal skills such as asking open questions, rather than non-verbal skills [38]. Although potential benefits were acknowledged by Shorey et al. [39], the challenges in building rapport and informal conversation skills were highlighted by participants. In their study of VPs to care for traumatized patients, participants sought a more user-friendly technology with the ability to ask their own follow-up questions, as the system was perceived as “*not easy to use*” [40]. These limitations suggest that technology alone is insufficient to develop the full spectrum of communication skills and therefore there is a need for additional pedagogical support.

In their systematic review, Lee et al. conclude that *“elaborate technology alone cannot guarantee effective learning*,* but evidence-based instructional interventions can facilitate its optimal use”* [36], and they suggest careful alignment of the VPs to interventions such as tutorials, human instructor feedback for debriefing, and team collaboration is required [36]. The contextual knowledge of students in our study is notable: they suggested the VPs be integrated into the curriculum as a preparatory exercise prior to meeting patients, demonstrating awareness of the limitations of the VPs with regard to real world transfer. This is consistent with previous work which recommends the use of VPs to complement other teaching modalities [29], as an adjunct to traditional teaching methods [41], prior to bedside learning [14, 42] or as part of wider group discussions [43].

Communication skills are complex, and best taught by experiential learning [36, 44], and VPs do appear to provide students with an immersive learning experience [45]. Feedback is central to their pedagogical value [22], helping students to understand “what good looks like” [28]. This is particularly important where VPs address emotionally challenging, or complex, consultations, as in this study. We attempted to embed structured feedback via the coach at each decision point; however, this feedback becomes pedagogically inadequate for the complex educational content of the VPs as it focuses on explaining correct/incorrect responses rather than addressing individual learning needs. The limited value of this feedback was evident from the intersection of Technological Pedagogical Content Knowledge domains as the technological requirement of this platform for predetermined, non-individualised, non-adaptive feedback meant that the students perceived it as inconsistent, didactic and limited. More widely in the literature, it appears that feedback within VP systems tends to be similarly one-directional, pre-recorded commentary rather than adaptive, individualised high-quality feedback addressing higher order thinking processes [27, 40, 46].

Lack of curricular integration and alignment with assessment impact student engagement with VPs [20, 28, 47]. Similarly, in the current study, contextual factors were evident in student reluctance to engage unless mandatory. Furthermore, within the VP itself, students elected not to engage with optional elements, regardless of whether tasks are perceived to be valuable. For example, reflection has been shown to be important to enrich the learning value of the VPs [20], and each VP in the current study offered a task that promotes self-reflection in order to achieve this. However, the autonomous navigation of the platform undermined pedagogical intent, and our results would indicate that deep levels of engagement and reflection did not happen. Stronger curricular integration including alignment with assessment, is required if VPs are to realise their potential and maximise educational outcomes.

### Study Limitations

This exploratory study was undertaken in a single institution, which limits generalisability beyond the study population. The study population is small and comprises medical student volunteers, which risks selection bias. TPACK was developed to assist educator integration of technology into their teaching for effective student learning, so our focus on the learner experience of that integration does extend beyond its intended purpose. Further, the misalignment of domains within our study may reflect platform-specific constraints rather than a limitation of VPs more broadly.

Despite these limitations, this study offers useful insights into student experiences of communication skills training, and highlights important considerations for future design and curricular integration.

## Conclusions

This exploratory study evaluated medical students’ perspectives of four virtual patient consultations through the TPACK lens. Our findings suggest that VPs support meaningful learning *about* communication for students through exposure to emotive, culturally complex and infrequently encountered clinical scenarios, enhancing confidence through scaffolded learning. However, our analysis of the data indicates a lack of alignment within and across TPACK domains. Within individual domains, students described communication learning occurring primarily at a cognitive and affective rather than performance level (Content), through modelling rather than skills practice (Pedagogy), while engagement is perceived as “clicking” rather than communicating (Technology). At the intersections of the domains, the misalignment becomes more apparent as although content and pedagogy align to build confidence through modelled best practice, the technology limits opportunity for authentic communication practice. Our findings indicate that autonomous navigation of this platform undermined pedagogical intent and this technology was unable to deliver valuable feedback. Context also affects engagement as students described the impact of lack of curricular integration and assessment alignment, a lack of capacity to undertake optional elements offered, regardless of educational value.

In conclusion, although VPs may usefully complement communication training for medical students when carefully integrated into the curriculum, their full potential will only be realised if VP technology evolves to more effectively support the pedagogical principles underpinning their design.

## Supplementary Information

Supplementary Material 1.

## Data Availability

The dataset for this study comprises transcripts from focus group and interview sessions and is available from the corresponding author on reasonable request.

## Abbreviations

VP: Virtual Patient consultation
RCSI: University of Medicine and Health Sciences in Ireland
TPACK: Technological Pedagogical Content and Knowledge

## References

[1] Pollak K, Alexander I, Tulsky SC, Lyna JA, Coffman P, Dolor CJ, Gulbrandsen RJ, Østbye P. Physician empathy and listening: associations with patient satisfaction and autonomy. J Am Board Fam Med. 2011;24(6):665–72. 10.3122/jabfm.2011.06.110025 PMC3363295 22086809

[2] Street R, Makoul L, Arora G, Epstein NK. How does communication heal? Pathways linking clinician–patient communication to health outcomes. Patient Educ Couns. 2009;74(3):295–301. 10.1016/j.pec.2008.11.015 19150199

[3] Roter D, Hall JA, Kern DE, Barker LR, Cole KA, Roca RP. Improving physicians’ interviewing skills and reducing patients’ emotional distress: a randomized clinical trial. Arch Intern Med. 1995;155:1877–84. 7677554

[4] Zolnierek K, DiMatteo B. Physician communication and patient adherence to treatment: a meta-analysis. Med Care. 2009;47(8):826. 10.1097/MLR.0b013e31819a5acc PMC2728700 19584762

[5] Stewart M. Effective physician-patient communication and health outcomes: a review. Can Med Assoc J. 1995;152(9):1423–33. PMC1337906 7728691

[6] Maguire P, Pitceathly C. Key communication skills and how to acquire them. BMJ Open. 2002;325(7366):697–700. 10.1136/bmj.325.7366.697 PMC1124224 12351365

[7] Evans R, Stanley J, Mestrovic RO, Rose R. Effects of communication skills training on students’ diagnostic efficiency. Med Educ. 1991;25:517–26. 10.1111/j.1365-2923.1991.tb00105.x 1758333

[8] Aspegren K. BEME Guide 2: Teaching and learning communication skills in medicine – a review with quality grading of articles. Med Teach. 1999;21:563–70. 10.1080/01421599978979 21281175

[9] Duffy F, Gordon D, Whelan GH, Cole-Kelly G, Frankel K, Buffone R. Assessing competence in communication and interpersonal skills: the Kalamazoo II report. Acad Med. 2004;79(6):495–507. 10.1097/00001888-200406000-00002 15165967

[10] Gilligan C, Powell M, Lynagh MC, Ward BM, Lonsdale C, Harvey P, James EL, Rich D, Dewi SP, Nepal S, Croft HA, Silverman J. Interventions for improving medical students' interpersonal communication in medical consultations. Cochrane Database Syst Rev. 2021;2(2):CD012418. 10.1002/14651858.CD012418.pub2. PMID: 33559127; PMCID: PMC8094582. PMC8094582 33559127

[11] Hargie O, Boohan M, McCoy M, Murphy P. Current trends in communication skills training in UK schools of medicine. Med Teach. 2010;32(5):385–91. 10.3109/01421590903394603 20423257

[12] Hoffman M, Ferri J, Sison C, Roter D, Schapira L, Baile W. Teaching communication skills: an AACE survey of oncology training programs. J Cancer Educ. 2004 Winter;19(4):220-4. 10.1207/s15430154jce1904_8. PMID: 15725639. 15725639

[13] Lanken P, Novack N, Daetwyler DH, Gallop C, Landis R, Lapin JR. Efficacy of an internet-based learning module and small-group debriefing on trainees’ attitudes and communication skills toward patients with substance use disorders: results of a cluster randomized controlled trial. Acad Med. 2015;90(3):345–54. 10.1097/ACM.0000000000000506 25295964

[14] Kononowicz A, Woodham A, Edelbring LA, Stathakarou S, Davies N, Saxena D, Car N, Carlstedt-Duke LT, Car J, Zary J. Virtual Patient Simulations in Health Professions Education: Systematic Review and Meta-Analysis by the Digital Health Education Collaboration. J Med Internet Res. 2019;21(7):e14676. 10.2196/14676 PMC6632099 31267981

[15] Kononowicz AA, Woodham L, Georg C, Edelbring S, Stathakarou N, Davies D, Masiello I, Saxena N, Tudor Car L, Car J, Zary N. Virtual patient simulations for health professional education. Cochrane Database Syst Rev. 2018 Jun 22;2018(6):CD012194. 10.1002/14651858.CD012194.pub2. PMCID: PMC6513573.

[16] Mishra P, Koehler MJ. Technological Pedagogical Content Knowledge: A Framework for Teacher Knowledge. Teachers Coll Record. 2006;108(6):1017–54.

[17] Koehler M, Mishra J. What is technological pedagogical content knowledge? Contemp Issues Technol Teacher Educ. 2009;9(1):60–70.

[18] Denniston C, Molloy E, Nestel D, et al. Learning outcomes for communication skills across the health professions: a systematic literature review and qualitative synthesis. BMJ Open. 2017;7:e014570. 10.1136/bmjopen-2016-014570 PMC5558817 28389493

[19] Noble L, Scott-Smith M, O’Neill W, Salisbury B. Consensus statement on an updated core communication curriculum for UK undergraduate medical education. Patient Educ Couns. 2018;101(9):1712–9. 10.1016/j.pec.2018.04.013 29706382

[20] Kelly S, Smyth E, Murphy P, Pawlikowska T. A scoping review: virtual patients for communication skills in medical undergraduates. BMC Med Educ. 2022;22(1):429. 10.1186/s12909-022-03474-9 PMC9166208 35659213

[21] Synthesia. [Available from: https://www.synthesia.io/

[22] Posel N, Fleiszer D, Shore BM. 12 Tips: Guidelines for authoring virtual patient cases. Med Teacher. 2009;31:701–8. 10.1080/01421590902793867 19513927

[23] Petko D, Mishra P, Koehler M. TPACK in context: An updated model. Computers Educ Open. 2025;8:100244.

[24] Braun V, Clarke V. Using thematic analysis in psychology. Qualitative Res Psychol. 2006;3(2):77–101.

[25] Stevens A, Hernandez J, Johnsen K, Dickerson R, Raij A, Harrison C, DiPietro M, Allen B, Ferdig R, Foti S, Jackson J, Shin M, Cendan J, Watson R, Duerson M, Lok B, Cohen M, Wagner P, Lind DS. The use of virtual patients to teach medical students history taking and communication skills. Am J Surg. 2006;191(6):806–11. 10.1016/j.amjsurg.2006.03.002 16720154

[26] Naveed M. The Value of Virtual Patients in Medical Education. Annals Behav Sci Med Educ. 2010;16:29–31.

[27] Isaza-Restrepo A, Gómez MT, Cifuentes G, Argüello A. The virtual patient as a learning tool: a mixed quantitative qualitative study. BMC Med Educ. 2018;18(1):297. 10.1186/s12909-018-1395-8 PMC6282259 30522478

[28] Botezatu M, Hult H, Fors UG. Virtual patient simulation: what do students make of it? A focus group study. BMC Med Educ. 2010;10:91. 10.1186/1472-6920-10-91 PMC3014956 21129220

[29] Plackett RKA, Mylan S, Kambouri M, Raine R, Sheringham J. The effectiveness of using virtual patient educational tools to improve medical students’ clinical reasoning skills: a systematic review. BMC Med Educ. 2022;22(1):365. 10.1186/s12909-022-03410-x PMC9098350 35550085

[30] Hoffmann T, Glasziou C, Boutron PP. Better reporting of interventions: template for intervention description and replication (TIDieR) checklist and guide. BMJ Open. 2014;348:g1687. 10.1136/bmj.g1687 24609605

[31] Ogrinc G, Armstrong GE, Dolansky MA, Singh MK, Davies L. SQUIRE-EDU (Standards for QUality Improvement Reporting Excellence in Education): publication guidelines for educational improvement. Acad Med. 2019;94(10):1461–70. 10.1097/ACM.0000000000002750 PMC6760810 30998575

[32] Huwendiek S, De leng BA, Zary N, Fischer MR, Ruiz JG, Ellaway R. Towards a typology of virtual patients. Med Teach. 2009;31:743–8. 10.1080/01421590903124708 19811212

[33] Kononowicz A, Zary A, Edelbring N, Corral S, Hege J. Virtual patients–what are we talking about? A framework to classify the meanings of the term in healthcare education. BMC Med Educ. 2015;15:11. 10.1186/s12909-015-0296-3 PMC4318546 25638167

[34] Jadoon M, Naushad K, Aman F, Khan Y, Durrani M, Ali S. Virtual Patients for Communication Skills Training: A Mixed Methods Evaluation. Cureus. 2025;17(8):e91005. 10.7759/cureus.91005 PMC12461232 41018381

[35] Ahn S, Jeong HW. Exploring nursing students’ experiences with virtual patient-based health assessment simulation program: A qualitative study. Nurse Educ Today. 2025;153:106826. 10.1016/j.nedt.2025.106826 40674991

[36] Lee J, Kim H, Kim KH, Jung D, Jowsey T, Webster CS. Effective virtual patient simulators for medical communication training: A systematic review. Med Educ. 2020;54(9):786–95. 10.1111/medu.14152 32162355

[37] Carmichael C, Mizrahi L. Connecting cues: The role of nonverbal cues in perceived responsiveness. Curr Opin Psychol. 2023;53:101663. 10.1016/j.copsyc.2023.101663 37572551

[38] Bearman M, Cesnik B, Liddell M. Random comparison of ‘virtual patient’ models in the context of teaching clinical communication skills. Med Educ. 2001;35(9):824–32. 10.1046/j.1365-2923.2001.00999.x 11555219

[39] Shorey S, Ang E, Ng ED, Yap J, Lau LST, Chui CK. Communication skills training using virtual reality: A descriptive qualitative study. Nurse Educ Today. 2020;94:104592. 10.1016/j.nedt.2020.104592 32942248

[40] Ekblad S, Mollica RF, Fors U, Pantziaras I, Lavelle J. Educational potential of a virtual patient system for caring for traumatized patients in primary care. BMC Med Educ. 2013;13(1):110. 10.1186/1472-6920-13-110 PMC3765390 23957962

[41] Carrard V, Bourquin C, Orsini S, Schmid Mast M, Berney A. Virtual patient simulation in breaking bad news training for medical students. Patient Educ Couns. 2020;103(7):1435–8. 10.1016/j.pec.2020.01.019 32019697

[42] Cook D, Triola A. Virtual patients: a critical literature review and proposed next steps. Med Educ. 2009;43(4):303–11. 10.1111/j.1365-2923.2008.03286.x 19335571

[43] Gonullu I, Bayazit A, Erden S. Exploring medical students’ perceptions of individual and group-based clinical reasoning with virtual patients: a qualitative study. BMC Med Educ. 2024;24(1):189. 10.1186/s12909-024-05121-x PMC10895817 38403641

[44] Van Merriënboer J, Kirschner JG. PA. Ten steps to complex learning: A systematic approach to four. -component instructional design: Routledge; 2017.

[45] Peddle MBM, McKenna L, Nestel D. Exploring undergraduate nursing student interactions with virtual patients to develop ‘non-technical skills’ through case study methodology. Adv Simul (Lond). 2019;4:2. 10.1186/s41077-019-0088-7 PMC6373120 30805205

[46] Jay R, Sandars J, Patel R, Leonardi-Bee J, Ackbarally Y, Bandyopadhyay S, Faraj D, O’Hanlon M, Brown J, Wilson E. The Use of Virtual Patients to Provide Feedback on Clinical Reasoning: A Systematic Review. Acad Med. 2025;100(2):229–38. 10.1097/ACM.0000000000005908 39485118

[47] Nowell L, Dolan S, Johnston S, Jacobsen M, Lorenzetti D, Oddone Paolucci E. Exploring Student Perspectives and Experiences of Online Opportunities for Virtual Care Skills Development: Sequential Explanatory Mixed Methods Study. JMIR Nurs. 2024;7:e53777. 10.2196/53777. PMID: 39167789; PMCID: PMC11375387. PMC11375387 39167789

