# Supplementary material for: Virtual patients and learning communication skills at “double speed”: a qualitative study using the TPACK framework
Source: Adv Simul (Lond). 2026 Aug 24;11:67. doi: 10.1186/s41077-026-00481-4 (PMC13615569; doi:10.1186/s41077-026-00481-4)
Supplement: Supplementary file 1 [file 41077_2026_481_MOESM1_ESM.docx]

**Appendix 1** Screenshot of platform depicting avatar coach and display of next options available to students
